# Thiol-Mediated Chemoselective Strategies for *In Situ* Formation of Hydrogels

**DOI:** 10.3390/gels4030072

**Published:** 2018-09-02

**Authors:** Jing Su

**Affiliations:** Department of Chemistry, Northeastern Illinois University, Chicago, IL 60625, USA; J-Su@neiu.edu; Tel.: +1-773-442-5773

**Keywords:** hydrogel, chemical crosslinking, chemoselective reaction, thiols

## Abstract

Hydrogels are three-dimensional networks composed of hydrated polymer chains and have been a material of choice for many biomedical applications such as drug delivery, biosensing, and tissue engineering due to their unique biocompatibility, tunable physical characteristics, flexible methods of synthesis, and range of constituents. In many cases, methods for crosslinking polymer precursors to form hydrogels would benefit from being highly selective in order to avoid cross-reactivity with components of biological systems leading to adverse effects. Crosslinking reactions involving the thiol group (SH) offer unique opportunities to construct hydrogel materials of diverse properties under mild conditions. This article reviews and comments on thiol-mediated chemoselective and biocompatible strategies for crosslinking natural and synthetic macromolecules to form injectable hydrogels for applications in drug delivery and cell encapsulation.

## 1. Introduction

Hydrogels are a class of highly hydrated materials with three-dimensional (3D) networks composed of hydrophilic polymers, which are either synthetic or natural in origin [1]. The structural integrity of hydrogels depends on the crosslinks formed between polymer chains via various physical interactions and chemical bonds. Because they have mechanical properties similar to the extracellular matrix in native tissues, hydrogels have been widely employed as implantable medical devices such as contact lenses and biosensors [2,3,4,5], surgical adhesives [6,7], immunoisolating capsules for tissue transplantations [8,9], scaffolds for tissue regeneration [10,11,12], and materials for drug delivery [13,14]. In particular, in situ forming hydrogels have been very attractive since they allow the delivery of polymer precursors in combination with cells and soluble drugs in aqueous solutions through injection, resulting in the formation of 3D functional hydrogel networks at desired locations [15,16]. 

Tremendous natural and synthetic materials have been developed for the in situ formation of physical hydrogels by noncovalent electrostatic attraction, hydrogen bonding, and hydrophobic interactions [15,17]. Many of these, however, need to be initiated by changes in pH, temperature, or ionic concentration, such as pH-sensitive leucine-zipper protein assembly [18], thermosensitive collagen gelation [19], Alginate-Ca^2+^ crosslinking [20], and peptide amphiphile assembly [21,22,23]. These environmental triggers are not always physiologically relevant or biocompatible, and can be irreversibly detrimental to encapsulated cells and macromolecule drugs. It is also difficult to reproducibly control these conditions in clinical settings. In addition, physically crosslinked hydrogels do not have sufficient mechanical strength and structural stability against environmental changes or even hydrodynamic shearing. On the other hand, the crosslinking of polymers through covalent chemical bond formation in physiological conditions can produce robust hydrogel networks bearing tunable mechanical strength and stability in a much greater range. Hydrogels formed in situ through chemical crosslinking alone or through a hybrid of physical and chemical crosslinking have been shown to meet the needs of many different biomedical applications, from artificial load-bearing connective tissue, to 3D tissue scaffolds, to controlled delivery of therapeutics [24,25].

**Figure 1:**
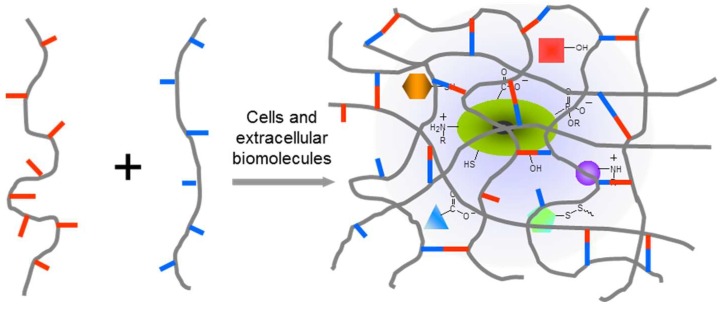
In situ crosslinking of hydrogels in the presence of the biological complex including cells, extracellular components, and therapeutic agents. Hydrogel networks should form upon chemoselective interactions between polymer precursors in order to minimize the disturbance to the biological systems under study.

In order to develop chemically crosslinked hydrogels to achieve a desired biomedical function, the right polymer precursors, crosslinking methods, and degradation properties of formed hydrogel are all essential. A good understanding of the biological system of interest is required to evaluate the interactions between the system and the applied polymer precursors, crosslinking catalyst/initiators, any possible product released from the crosslinking reaction, and degradation products from hydrogels. In many cases, methods of polymer crosslinking would benefit from being highly selective to avoid cross-reactivity and adverse effects on functional components of the biological system (Figure 1). In physiologically relevant environments, as focused on in this review, chemoselectivity is defined as the preferred reactivity of a chemical group toward another specific functionality in the presence of multiple potentially reactive functionalities, especially those existing in biological complexes. The past two decades have witnessed a remarkable advancement of bio-orthogonal chemical reactions that covalently connect unnatural chemical structures [26], for example, 1,3-dipolar click cycloaddition and Diels–Alder cycloaddition, providing promising solutions to eliminate interference with biological systems during the formation of polymeric hydrogels, as summarized in several recent reviews [24,25,27]. However, these unnatural, usually expensive building blocks may significantly increase the cost of materials, and this limits the use of bio-orthogonal reactions for producing hydrogels in reality.

On the other hand, polymers presenting naturally existing functionalities such as the amino groups (NH_2_) and thiols (SH, sulfhydryl), are still widely used in biomedical research and applications because of the relatively low cost and great availability. For example, the natural polymer chitosan presents amino groups; polypeptides can present amino groups through lysine residues and thiol groups at cysteine residues; and synthetic macromolecules functionalized with amine or thiol groups are readily available from many chemical suppliers at affordable prices. When these polymers are used in the presence of biological components, a commonly applied strategy for achieving chemoselectivity during hydrogel crosslinking is by kinetic control, in which exogenous polymer precursors are applied at much higher concentrations than those biological components that are potentially reactive, driving crosslinking reactions to occur mainly between externally supplied polymers. 

Compared to the amino group, the thiol group occurs at lower abundance in naturally existing molecules and therefore bears relatively higher chemoselectivity, which can be kinetically “manipulated” to direct reactions mainly taking place between exogenous thiol-containing polymers and crosslinkers. This review summarizes the strategies for chemically crosslinking polymers through thiol-mediated reactions to form hydrogels in situ for biomedical applications. The goal of this review is not to provide a complete coverage of works that utilized these strategies, but rather to highlight inspiring research that opened new horizons of chemoselective hydrogels. Discussions are aimed at giving guidance on how the crosslinking method shall be chosen based on the type of polymer precursors, the desired gelation kinetics, the need for modification of hydrogel networks with biochemical cues, and the effects of crosslinking on the biological targets and surroundings.

## 2. Reactivity of the Thiol Group

A thiol compound refers to an organic molecule containing a sulfhydryl (SH) group bonded to a carbon atom. The SH group is a naturally existing functionality, as seen on the side chain of the amino acid cysteine in proteins and peptides, and in low-molecular-weight natural molecules [28]. The SH group participates in the formation of disulfide bonds (S–S) that are essential to tertiary structures of proteins. This functionality or its anion form (–S^−^) is also present in the active sites of many enzymes such as caspase proteases and ubiquitin-conjugating enzymes [29,30], using its nucleophilicity to mediate various transformations of substrates. These natural biochemical reactions inspired the development of thiol-based conjugation methods, mainly utilizing the good nucleophilicity of the sulfur atom toward electron-deficient moieties including α-halo carbonyl-containing compounds and α, β-unsaturated compounds, such as maleimide and vinyl sulfone, as reviewed by Stenzel [31]. At the physiological pH, the nucleophilicity of thiol is 1000 times stronger than the ionized amino group, enabling thiols to react with electrophiles with a much higher selectivity. Therefore, the thiol group has been an attractive functionality used in the chemical modification and crosslinking of polymers [31], despite the strong odors of thiol-containing compounds. 

Most of the thiol-engaged crosslinking methods can be assigned to two categories according to the mechanisms of reactions: addition reactions and substitution reactions. Addition reactions simply connect the reactive groups present on the polymers (or crosslinkers) without releasing any side products to the solution environment. Some of the most widely used bioconjugation methods including Michael-type addition and photoinitiated thiol-ene addition belong to this category. In substitution reactions each bonding formation is accompanied by the release of by-products, usually low-molecular-weight molecules. Several reactions for the formation of disulfide bonds, native chemical ligation and the thiol-epoxy reaction are examples of thiol-involved substitution reactions. Presumably, addition reactions would be ideal for the crosslinking of biocompatible polymeric molecules because no side products are generated and interfere with the biological targets in contact with the hydrogels formed. On the other hand, the use of substitution reactions for in situ hydrogel formation does require additional examination of how released side products affect a system of study, such as encapsulated drugs and cells/tissues. Methods based on both types of reaction mechanisms have been applied in the development of in situ forming hydrogels. Table 1 outlines the pros and cons of each crosslinking reaction that will be discussed in detail in Section 3 and Section 4.

## 3. Substitution Reactions of Thiols for Hydrogel Crosslinking

### 3.1. Formation of Disulfide Bonds

**Figure 2:**
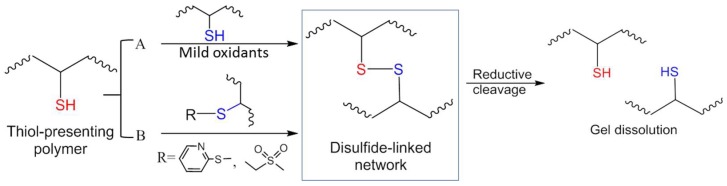
Strategies of disulfide bond formation for in situ crosslinking of polymer hydrogels. (**A**) Mild oxidation of thiols. (**B**) Thiol-disulfide exchange (a substitution reaction). The disulfide-crosslinked network can break down when treated with reductive agents (e.g., soluble thiols).

Figure 2 summarizes the strategies for constructing disulfide-crosslinked hydrogels. Traditionally disulfide formation results from the oxidation of thiols exposed to molecular oxygen in ambient air or mild oxidizing reagents such as Cu(II)SO_4_ (Figure 2A). Disulfide bonds can dissociate in a reductive environment, for example, in the presence of free thiols such as glutathione (GSH) and dithiothreitol (DTT), which allows the design of redox-responsive degradation [32,33]. 

**Figure 3:**
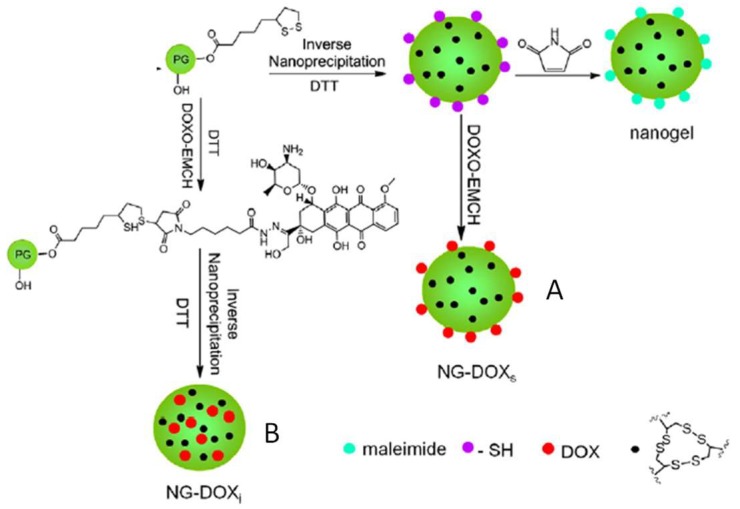
Hydrogel nanoparticles generated by the crosslinking of PEGs terminated with reduced dithiolane that can form disulfide bonds. Maleimide-tagged doxorubicin can be attached either at the surface of the gel particles (**A**) or in the interior (**B**) depending on the order of gel crosslinking and drug conjugation. Image modified with permission from [34]. Copyright 2014 Elsevier.

The formation of disulfide-crosslinked hydrogel nanoparticles was reported by Zhang et al. using hyperbranched polyglycerol terminated with reduced dithiolane groups (Figure 3) [34]. In a stepwise manner, thiol groups on the polymers were partially used for disulfide crosslinking, while the remaining thiols were reserved to conjugate maleimide-tagged doxorubicin (DOX). By carrying out the gel formation and DOX conjugation in different orders, the researchers were able to control the presentation of the DOX drug molecules either at the gel surface or within the gel particles. Both types of nanogel particles were shown to be redox-degradable through disulfide cleavage and tested for delivering the DOX to cancer cells in vitro. 

Because a strong oxidative environment can damage the function of many bioactive molecules, mild oxidative conditions are needed to crosslink thiol-presenting polymers to form disulfide hydrogels for encapsulating drugs and cells. However, most of these mild oxidation conditions cannot produce a sufficiently crosslinked network in a short period of time (usually it needs more than a day) [35]. The slow reaction kinetics allows undesired interactions between the thiol-presenting polymers/crosslinkers and those naturally occurring thiols, disulfides and electrophiles to become competitive, which may alter the biological system of study [36]; therefore, this crosslinking method is not always considered as biocompatible. Additionally, the slow crosslinking prevents disulfide hydrogels from applications in clinical settings where the rapid gelation of injectable materials is necessary. 

**Figure 4:**
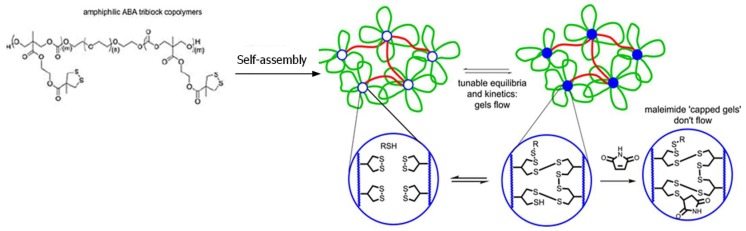
Crosslinking of dithiolane-containing polymeric micelles by thiol-triggered disulfide-thiol exchange to form self-healing hydrogels. The addition of a thiol-capturing compound, maleimide, decreased the amount of free thiols necessary for the disulfide-thiol exchange, changing the dynamic gel to a permanent network. Image modified with permission from [39]. Copyright 2017 ACS.

Thiol-disulfide exchange is a substitution reaction used by nature to form disulfide bonds (Figure 2B); however, it also occurs at a relatively slow rate unless high local concentrations of thiols and disulfides are available [37,38]. Zhang and Waymouth recently reported a thiol-triggered ring-opening of thiolane disulfide in polymeric self-assembly produced free thiol groups, which participated in the formation of a dynamic thiol-disulfide exchange hydrogel network with a self-healing property (Figure 4) [39]. Maleimide was added to consume free thiol groups within the hydrogel and prohibited thiol-disulfide exchange, changing an adaptable network to a rigid, permanent network. 

**Figure 5:**
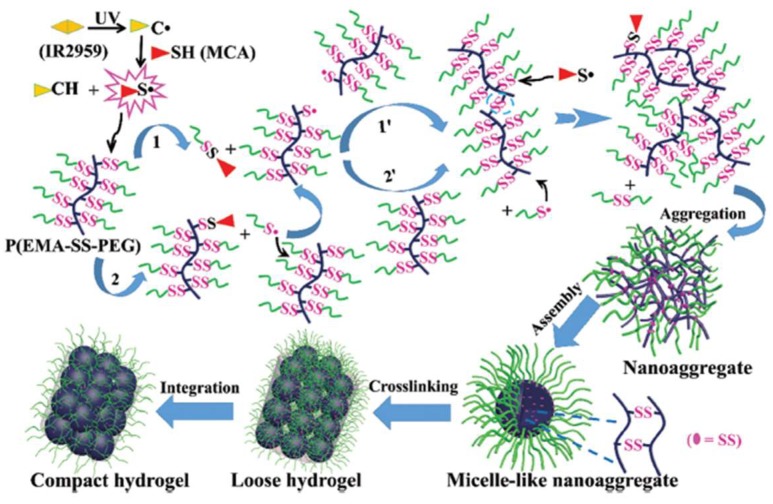
The mechanism of UV-triggered thiol-disulfide exchange for the formation of hydrogels. Image reproduced with permission from [40]. Copyright 2016 RSC.

A photo-initiated, radical-mediated thiol-disulfide exchange reaction was demonstrated by Wang et al. to construct hydrogels using a comb-like polymer of polyethylene glycol grafted with disulfide-linked poly(ethyl methacrylate) derivative P(EMA-SS-PEG) [40]. UV irritation in the presence of a radical initiator triggered the breakage of some disulfides between PEG and grafted EMA and generated free thiols that formed new disulfides among PEG polymer chains to produce crosslinked network through a mechanism suggested in Figure 5.

**Figure 6:**
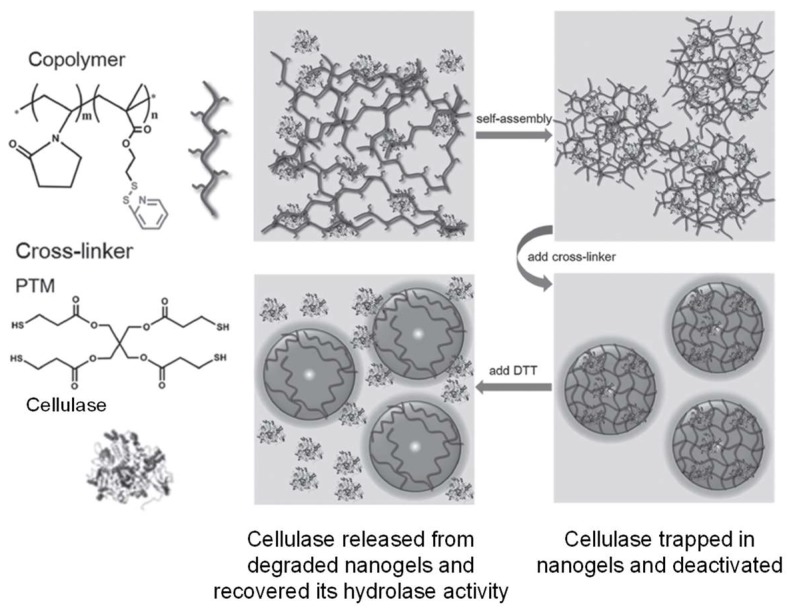
Crosslinking of pyridyl disulfide-presenting polymer self-assembly with thiol-terminated linkers for the encapsulation and deactivation of a cellulase. Image modified with permission from [42]. Copyright 2016 Wiley.

A modified version of thiol-disulfide exchange between thiols and activated disulfides (e.g., pyridyl disulfide) proceeds faster than thiol oxidation to form disulfides, and has been applied to polymer crosslinking to form nanogels within hours [41]. Recent examples include the work by Peng et al. that demonstrated the crosslinking of pyridyl disulfide-presenting copolymers with four-armed, thiol-terminated linkers [42]. The disulfide-crosslinked nanogels were used for the encapsulation and deactivation of a cellulase. The enzyme activity of the cellulase was recovered as the protein was released from the hydrogel trap upon DTT-induced disulfide bond breaking and gel degradation (Figure 6).

**Figure 7:**
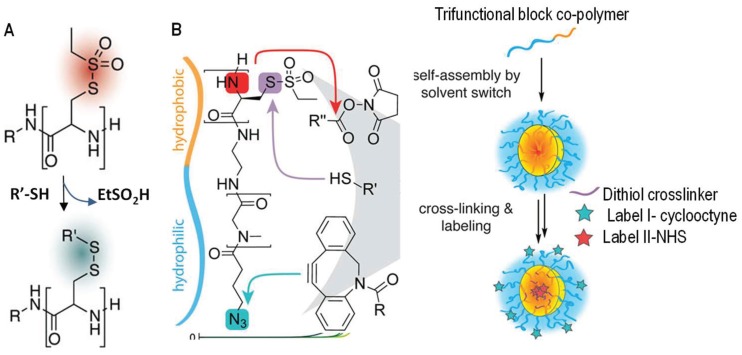
Thiol-disulfide exchange between thiols and thiolsulfonates for the functionalization (**A**) and chemical crosslinking (**B**) of thiol-functionalized polymers. In both cases the formed disulfide links are redox-responsive. This exchange reaction in (**B**) was shown to be orthogonal to ester formation and 1,3-dipolar cyclic addition used for labeling the gel particles with dyes. Images are modified with permission from [44]. Copyright 2017 ACS.

A rapid exchange between thiols and thiosulfonates was reported by Schäfer and co-workers [43]. Polyamides presenting *S*-ethylsulfonyl-l-cysteine units were used to react with free thiols and generate disulfide tags along the polymer chains within a minute (Figure 7). This substitution reaction was shown to be quantitative and highly selective, and released small alkylsulfonate as a side product with low toxicity. This method has been used by the same research group to react thiolsulfonate-containing block copolymers with dithiol crosslinkers to interconnect self-assembled micelles (Figure 7B) [44], and may serve as an effective strategy for the production of disulfide hydrogel networks in situ for other applications.

### 3.2. Native Chemical Ligation

**Figure 8:**
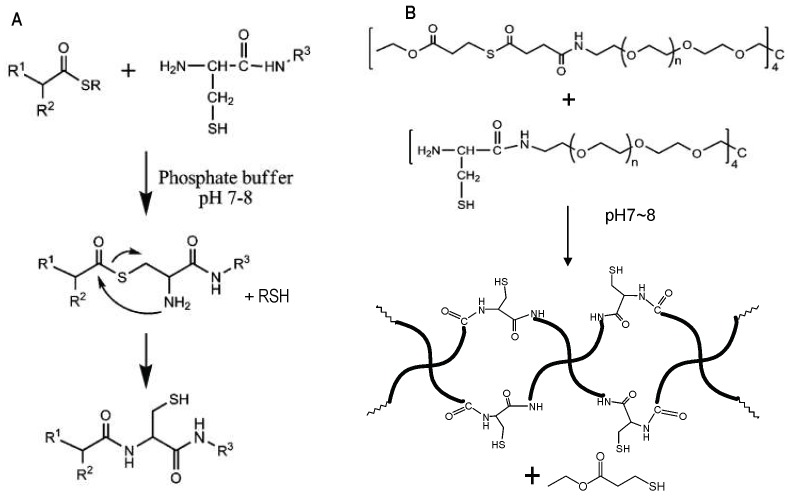
(**A**) A general scheme of NCL. (**B**) Catalyst-free in situ hydrogel crosslinking by native chemical ligation. Four-armed PEG macromonomers terminated with *N*-terminal cysteine and thioester groups crosslink to form gels in aqueous media at pH 7~8. Image reproduced with permission from [35]. Copyright 2009 ACS.

Native chemical ligation (NCL) is the substitution reaction between a thioester and a 2-aminoethanethiol moiety, for example, an *N*-terminal cysteine residue of a protein or peptide. The reaction yields an initial thioester exchange product that spontaneously undergoes an *S*- to *N*-acyl migration to form a new amide bond [45], as shown in Figure 8A.

The chemoselectivity of NCL lies in that only the 2-aminoethanethiol moiety is reactive with a thioester to complete this transthioesterification-rearrangement process and form a stable amide bond. A thiol group alone, as on the side chain of a cysteine residue in the middle of polypeptide chains, or an amino group only, does not interfere with the NCL reaction. This mild ligation method has proven useful in the chemical synthesis of large peptides and proteins [46] and peptide-based block polymers and dendrimers [47,48]. In an early application of NCL to covalently connect polymers, Collier and co-workers reported the cross-linking of pre-assembled *β*-sheet-forming peptides with terminal thioester and cysteine groups to increase the stiffness of physical peptide hydrogels [49].

Hu et al. applied NCL to form PEG hydrogels in situ in an aqueous environment (Figure 8B) [35]. Mixing solutions of four-armed PEG macromonomers terminated with thioester and cysteine groups at pH 7~8 resulted in the formation of robust hydrogels rapidly within minutes. These hydrogels remained stable after treatment with excess reducing agents such as tris(2-carboxyethyl)phosphine (TCEP) and 2-mercaptoethanol, implying the polymeric network was crosslinked by amide bonds formed through the NCL mechanism, rather than by intermolecular disulfide bonds between Cys groups. This hydrogel formation strategy was later applied to the encapsulation of extracellular matrix proteins to engineer the microenvironment of human mesenchymal stem cells in 3D culture by Jung and co-workers [50]. In a more recent application of the strategy, cysteine-functionalized hyaluronic acids were crosslinked with multi-armed PEG-thioester macromonomers to form hydrogels in situ, expanding the choices of biomedical polymers for NCL crosslinking [51].

**Figure 9:**
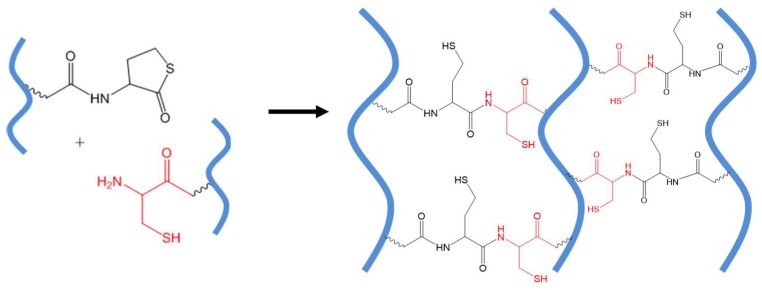
The native chemical ligation (NCL) reaction between polymers presenting thiolactone (a cyclic thioester) and a 2-aminoethanethiol group does not release any soluble thiol by-product as in the traditional NCL reactions. All thiols groups are immobilized onto the covalent network of crosslinked polymers.

Early studies of NCL-mediated hydrogel crosslinking revealed some drawbacks of the method including the instability of thioesters toward hydrolysis and especially a potential concern about the adverse biological effects of low-molecular-weight thiol side products released from NCL and hydrolysis of thioesters, which may vary in different applications. The use of thiolactone in place of a thioester to react with cysteine can generate an amide bond without releasing a soluble thiol by-product, as shown in Figure 9. Fan and co-workers used thiolactone-grafted and cysteine-grafted poly(glutamic acid) precursors to form NCL-crosslinked hydrogels compatible with cultured mouse fibroblast cells [52].

**Figure 10:**
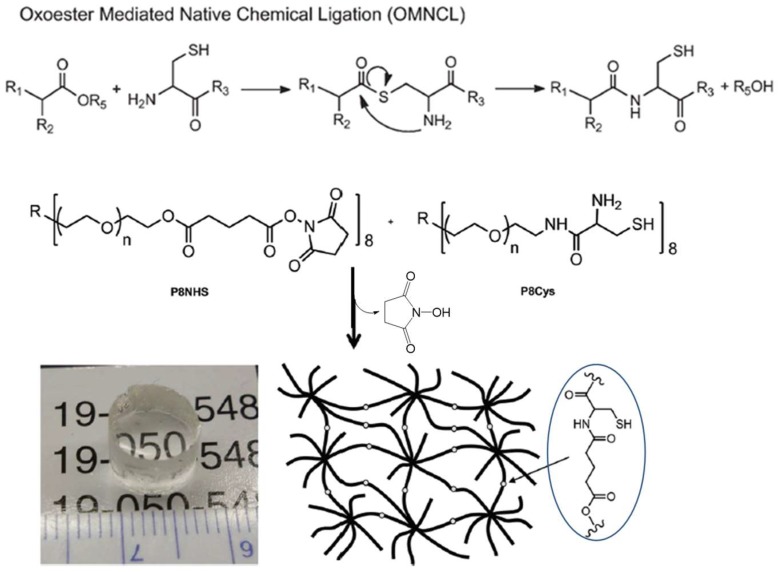
A generalized reaction scheme for oxo-ester-mediated native chemical ligation (OMNCL) used for crosslinking eight-armed PEG macromonomers to form a chemical hydrogel network. Image reproduced with permission from [53]. Copyright 2013 RSC.

A modified version of the NCL method, oxo-ester-mediated NCL (OMNCL, Figure 10), was used by Strehin and co-workers to crosslink eight-armed PEG macromonomers terminated with *N*-hydroxysuccinimide (NHS) active ester and cysteine [53]. The gelation of these macromonomers occurred rapidly within 20 s after mixing, while the crosslinking of macromonomers presenting NHS and amino groups was shown to be 10 times slower, confirming the initial, rapid ester exchange between NHS and the thiol group in cysteine, followed by S→N rearrangement to form an amide bond. Although NHS ester is hydrolytically unstable, the hydrolysis product, NHS, also as the side product released from the OMNCL, has much less interference with biological systems than thiols produced in NCL. In a recent report by Boere et al., NCL and OMNCL methods were compared in connecting cysteine-grafted poly(*N*-isopropylacrylamide) with linkers containing thioester or NHS [54]. Chemical crosslinking by OMNCL produced hydrogels with higher stiffness and lower in vitro toxicity to mouse endothelial cells than gels formed by NCL. 

**Figure 11:**
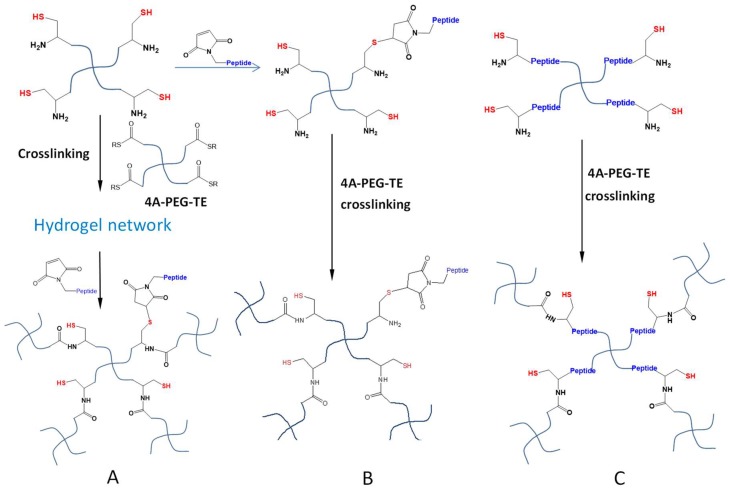
Functionalization of NCL hydrogels. (**A**) The hydrogel network crosslinked by the NCL reaction presents thiols that can react with maleimide-attached peptides (or other bioactive molecules) post-gelation. (**B**) Maleimide-attached molecules can be conjugated to thiol-polymers prior to NCL crosslinking. (**C**) Molecules of desired properties can be synthetically inserted into the thiol-polymers or thioester-polymers to form functional NCL hydrogels.

Hydrogel crosslinking by NCL and OMNCL reactions generates free thiol moieties bound to the polymeric network, which may introduce anti-oxidative properties to hydrogels and therefore can increase the survival of cells cultured in the hydrogel or at the gel surface [55]. Furthermore, these immobilized thiol groups can be used to conjugate molecules containing thiol-reactive structures including maleimide and α-halo carbonyl groups, to install desired functions in formed hydrogel networks (Figure 11A) [35]. A second strategy of hydrogel functionalization is to sacrifice a certain amount of thiol groups in the polymer precursors to attach thiol-reactive molecules prior to in situ gelation. Su et al. utilized a rapid and quantitative Michael-type addition to conjugate a maleimide-terminated, anti-inflammatory peptide to a low percentage of Cys moieties of four-armed PEG-Cys macromonomers (Figure 11B) [56]. Subsequent mixing of the modified macromonomers with thioester-terminated four-armed PEGs resulted in the formation of peptide-conjugated NCL hydrogels that promoted the survival of encapsulated pancreatic islet β-cells. However, a limitation of this functionalization strategy is that bioactive molecules can only be incorporated at low densities since the majority of Cys groups need to be retained to react with thioester groups for productive crosslinking. Alternatively, the incorporation of the functional molecules (e.g., cell survival-promoting peptides) into the initial macromonomer structures for NCL or OMNCL crosslinking (Figure 11C), may offer a better way to present these molecules at more variable densities in a hydrogel system.

**Figure 12:**
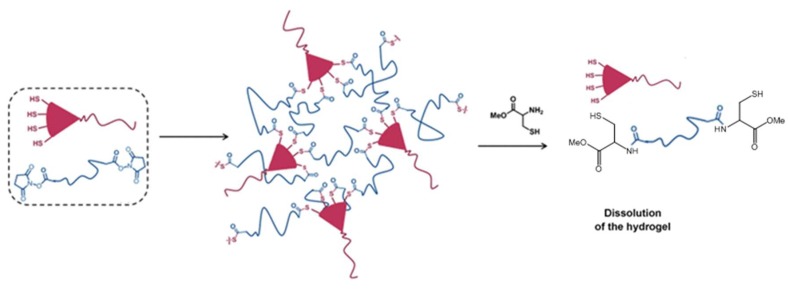
The use of NCL to dissolve a thioester-linked hydrogel network by the addition of a cysteine derivative. Image modified with permission from [57]. Copyright 2013 Wiley.

An interesting application of the NCL strategy in the dissolution of thioester-linked hydrogels was demonstrated by Ghobril and co-workers [57]. Dendritic macromers presenting multiple thiol termini were used to react with a PEG crosslinker containing the NHS active esters to form thiolester networks (Figure 12). A small thiol molecule, methyl ester of l-cysteine, added at high concentrations to the hydrogel, was able to trigger the dissolution of the gel by the NCL mechanism that broke the thioester links in the macromolecular network. This gel dissolution strategy was applied to the development of dissolvable sealant for wound closure [57,58], as examples of smart biomaterial design inspired by good understanding of chemical reaction mechanisms.

### 3.3. Thiol-Epoxy Reaction (Ring-Opening Reaction of Epoxides)

**Figure 13:**
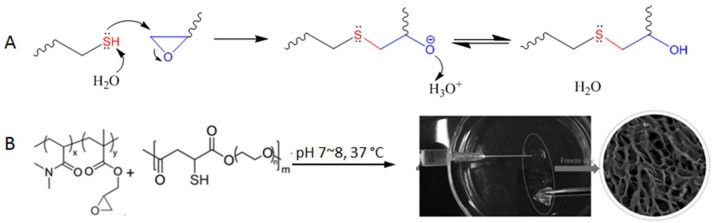
(**A**) Thiol-triggered ring opening of epoxides undergoes a nucleophilic substitution mechanism but does not produce byproducts. (**B**) The thiol-epoxy reaction for the crosslinking of polymers to form injectable hydrogel. Image modified with permission from [61]. Copyright 2016 Wiley.

The thiol-epoxy reaction involves a nucleophilic substitution between the thiol/thiolate nucleophile and an electrophilic carbon on the epoxy ring, leading to the ring opening followed by proton transfer to generate a thioether-alcohol product (Figure 13A). Although initiated by the substitution mechanism, the overall outcome of the reaction is similar to an addition reaction in which there are no organic by-products.

The thiol-epoxy reaction has been used for the polymerization of monomer building blocks [59], the functionalization of polymers [60], and was shown by Gao et al. for crosslinking poly(*N*,*N*-dimethylacrylamide-co-glycidyl methacrylate) to form hydrogels in situ under physiological conditions (pH 7~8, 37 °C, Figure 13B) [61]. The polymer precursors started gelation within seconds when present at 10~20% weight percent in aqueous solutions, and the resultant hydrogels were shown to be nontoxic to Hela cells incubated together. The fast reaction kinetics and relatively high selectivity shown in this study suggest a strong potential of the thiol-epoxy reaction for the development of injectable hydrogel materials.

## 4. Addition Reactions Involving Thiols for Hydrogel Crosslinking

### 4.1. Michael-Type Additions

**Figure 14:**
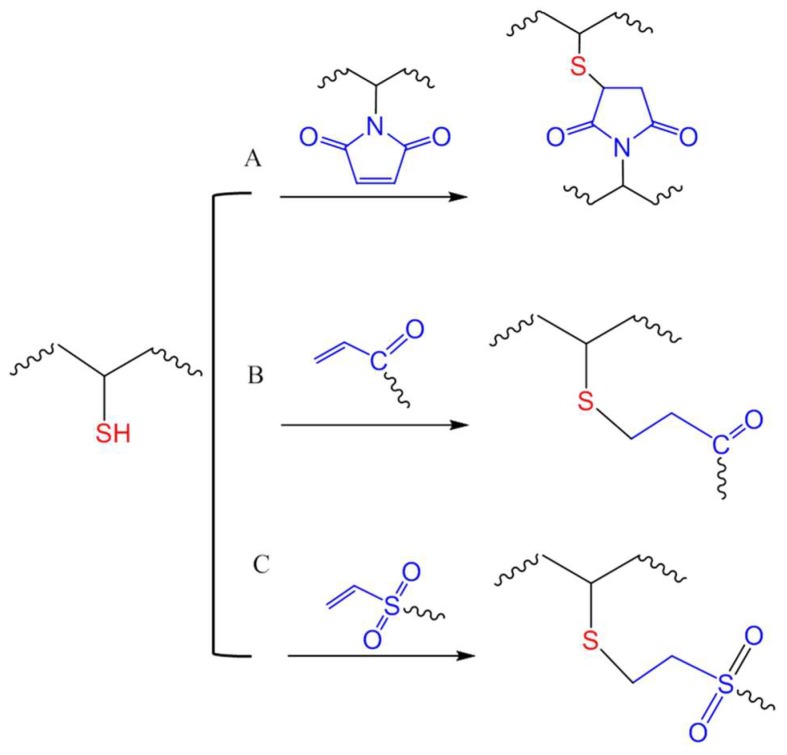
Michael-type additions for in situ crosslinking of polymers through reactions of thiols with maleimide (**A**), acrylate (**B**), or vinyl sulfone (**C**) groups.

A Michael-type addition reaction refers to the addition of a nucleophile to an electron-deficient carbon–carbon double bond (olefin). This type of reaction between thiols and various activated double bonds, such as those in acrylates, maleimide and vinyl sulfones (Figure 14), has been widely applied in bioconjugation and biomaterials due to mild conditions and high yields. One of the early examples of thiol-acrylate Michael addition for hydrogel crosslinking was reported by Hubbell et al., where PEGs multi-functionalized with thiols and acrylate groups were crosslinked to form hydrogels for the encapsulation and controlled release of albumin [62]. The same strategy was used by Elia and co-workers to produce hydrogels by connecting thiol-modified heparin and hyaluronic acid with PEG-diacrylate for the local delivery of growth factors by injection to achieve stimulated angiogenesis in a mouse model [63]. In a very recent application of this reaction, four-armed PEG-acrylate macromonomers were crosslinked by thiol groups of cysteines in heparin-binding peptides to generate a 3D environment for culturing encapsulated mouse cardiac stem cells [64]. The study showed that the crosslinking kinetics and the mechanical and biodegradation properties of the hydrogel can be tuned by varying the concentrations of the acrylate-macromonomers and the cysteine-bearing peptides to resemble the natural mouse heart tissue. 

While maleimide is an electron-deficient olefin widely used for thiol-mediated bioconjugation [31,65], its utilization in chemical crosslinking of hydrogel has not been as common as acrylates and vinyl sulfones. Phelps et al., demonstrated that in triethanolamine-buffered solutions, four-armed PEG macromonomers presenting maleimide groups were able to crosslink by reacting with peptides presenting two terminal cysteines with faster kinetics compared to acrylate- or vinyl sulfone-presenting four-armed PEG macromere [66]. However, vinyl sulfone moieties have been shown in other reports as the more reactive in Michael-type additions for the crosslinking of PEG hydrogel networks [36,67]. Vinyl sulfones are expected to merely undergo nucleophilic addition at the olefin double bond, while the addition to maleimide is often accompanied by a ring opening reaction that generate charged products that may alter the properties of polymers after crosslinking [68]. 

A drawback of the Michael-type addition for hydrogel crosslinking manifests where nucleophilic molecules other than thiol-containing polymers/linkers are present during gel formation. Hammer et al. investigated the nonspecific chemical interaction between lysosome and linear PEGs terminated with maleimide, acrylamide or vinyl sulfone [36]. Elevating the pH of the environment (inorganic buffers) from 4 to 9 resulted in increased PEG conjugation to lysozyme by each of the three groups tested, with vinyl sulfone giving the highest modification and acrylamide the lowest modification of the protein. Even when high concentrations of thiol-functionalized PEGs were introduced to compete with lysozyme to react with these electron-deficient olefin groups, protein modification through the vinyl sulfone was still significant, implying a relatively low chemoselectivity of vinyl sulfone. Additionally, these fast Michael-type additions may not be ideal for applications that need consistent hydrogel crosslinking. For example, nonuniformity at the microscale due to different degrees of crosslinking were observed in PEG hydrogels formed by the thiol-maleimide addition, resulting in varied cellular responses to hydrogels, as reported by Darling et al. [69].

**Figure 15:**
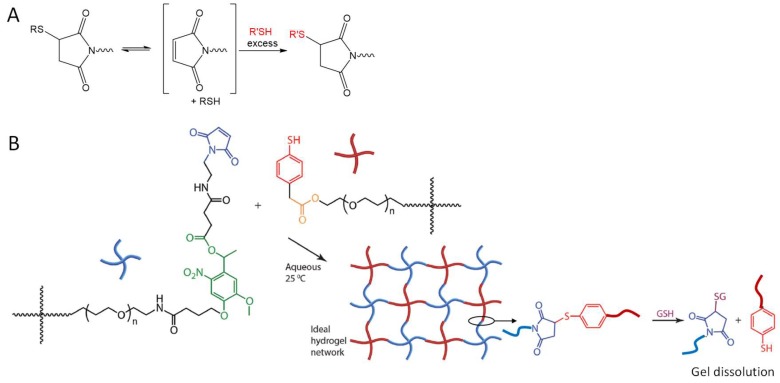
Retro Michael-type addition (**A**) can be utilized to break down networks of crosslinked polymers on demand (**B**) in the presence of soluble thiols (e.g., glutathione, GSH), as an orthogonal method to the degradation by light (the reactive group shown in green) and hydrolysis (the reactive group shown in orange). Image B modified with permission from [71]. Copyright 2015 RSC.

The dynamic character of Michael-type addition reactions has received increasing attention because of its potential use in the development of degradable and malleable polymeric hydrogel materials (Figure 15A). Baldwin and Kiick reported maleimide-thiol crosslinked hydrogels degraded through a glutathione-triggered retro Michael-type addition reaction, which took place at a slower rate than gel breakdown by reductive cleavage of disulfides [70]. A similar strategy of crosslinking and dissolution of hydrogels through the reversible maleimide-thiol addition was later utilized by Kharkar and co-workers to develop multifunctional PEG hydrogels that degraded in response to three orthogonal stimuli, one of which is reductive GSH that triggers the retromaleimide-thiol addition by, with tunable degradation kinetics (Figure 15B) [71]. Kuhl et al. and Chakma et al. reported that the reversible Michael-addition crosslinking could be used to improve the self-healing properties of gels at elevated temperature (≥60 °C) and increased pH (≥9) [72,73]; however, these conditions may not find immediate application in current biomedical studies. 

### 4.2. Photo-Initiated Thiol-Ene and Thiol-Yne Additions 

Photo-initiated crosslinking of hydrophilic polymers is one of the most widely used methods for in situ hydrogel formation. Traditionally, a solution containing polymeric precursors with photo-reactive groups (e.g., acrylate derivatives) and low concentrations of radical-generating initiators is delivered to a desired location, and subsequent irradiation of UV light triggers the crosslinking of polymers to form gels [74,75,76,77]. Although, in principle, photocrosslinking can take place preferably among reactive polymers through kinetic control, the reaction lacks high chemoselectivity because many bioactive molecules contain UV-sensitive groups and can be chemically transformed upon UV irradiation. There are inherent limitations associated with the photo-curing process such as the use of potentially toxic photoinitiators [78,79], the generation of highly reactive radicals, and the eventual exothermic effect of photo-reactions, which can be detrimental to functions of biological systems in contact with hydrogels [80]. Limited penetration of light into deeper tissues further restricts the use of photocrosslinked hydrogels as carriers of therapeutics in many applications [74]. Nevertheless, the great availability of various polymers with photo-reactive groups and the convenience of applying light on demand to control gelation and thus the mechanic properties of the hydrogels render this method still attractive.

**Figure 16:**
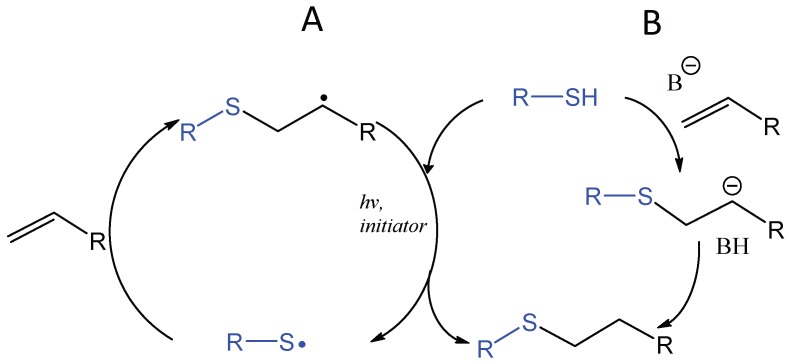
Photoinitiated, radical-mediated thiol-ene addition generates a covalent bond rapidly between a thiol-presenting compound and an alkene (**A**). When electron-deficient alkenes are used, the photoinitiated thiol-ene reaction is often accompanied by the base-catalyzed Michael-type addition reaction (**B**).

The application of thiol addition across unsaturated carbon-carbon bonds, such as the double bonds in alkenes and triple bonds in alkynes, has flourished in the field of biomaterials in the last decade [81,82,83,84]. The photochemically induced thiol-ene reaction proceeds by a radical mechanism to give an anti-Markovnikov-type thioether, which is compatible with water and oxygen and usually accompanied by Michael-type addition (Figure 16). An early study by Roydholm et al. described that varying the ratios of thiol- and acrylate-containing polymer precursors and reaction conditions could switch the main hydrogel crosslinking mechanism between the different modes, Michael-type addition, radical-initiated thiol-ene, or the mixed mechanism, resulting in hydrogels with adjustable mechanical and degradation properties [85]. The photochemical thiol-ene addition has been considered “clickable” because of its high efficiency and orthogonality to a wide range of functional groups [86,87]. Beside its use in bioconjugation [88,89,90], this reaction has been widely applied to crosslinking of polymeric hydrogels for the delivery and controlled release of therapeutics as discussed in a recent review [91].

**Figure 17:**
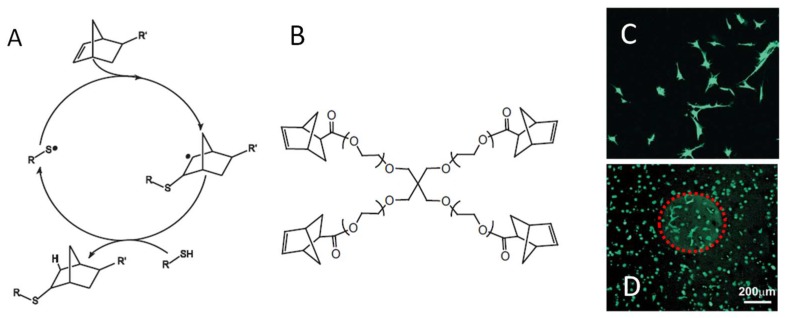
(**A**) A general mechanism of radical-mediated thiol-norbornene addition. (**B**) Four-armed norbornene-presenting PEG macromonomers were used to crosslink with di-cysteine-terminated peptides to form hydrogels. The gel can be modified uniformly with a cell adhesion peptide CRGDS during crosslinking (**C**) or after gelation through unreacted norborenene at defined locations shown as the red sphere in (**D**), both supporting cell attachment and spreading. Images reproduced with permission from [92]. Copyright 2009 Wiley.

The use of norbornene as the ene moiety enables a strain-promoted thiol-ene reaction and further improves the gelation speed and chemoselectivity of this method (Figure 17A) [77,92]. Shih et al. reported a comparison between hydrogels formed by thiol-ene photocrosslinking of PEG-tetra-norbornene (PEG4NB) and dithiothreitol (DTT) and those by Michael addition crosslinking of PEG-tetra-acrylate with DTT [93]. The thiol-ene photocrosslinking resulted in faster gelation rate and higher degree of network formation as revealed by rheometry. Fairbanks et al. demonstrated the functionalization of thiol-norbenene photocrosslinked hydrogels in two ways: (1) dicysteine-terminated peptides containing a protease substrate sequence were used to react with norbornene-terminated crosslinkers to form degradable hydrogels; (2) the conjugation of cysteine-containing peptides to the formed gels were achieved through a second thiol-ene reaction between the cysteine thiol and unlinked norbornene on the polymer network [92]. Both methods were used to present an RGD-presenting cell adhesion peptide on hydrogels to support the attachment and spreading of human MSC cells (Figure 17B). The majority of studies using thiol-norbornene linked hydrogels for the delivery of therapeutics have been reviewed elsewhere [77,91]. Recent advancement in the fast-booming regime of these hydrogels includes varying the type of polymers, radical initiators [94,95], and light sources [95,96] combined with modern material processing technologies [97,98,99], providing better control over the mechanical and biological properties of thiol-ene hydrogels for biomedical applications.

**Figure 18:**
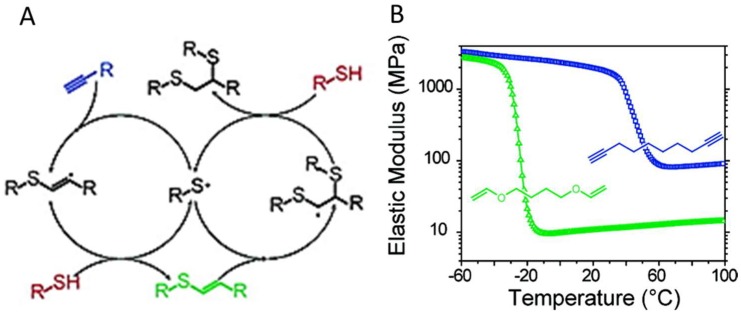
(**A**) A general mechanism of photoinitiated, radical-mediated thiol-yne addition. (**B**) The crosslinking between four-armed C(CH_2_OOCCH_2_CH_2_SH)_4_ and a dialkyne linker produced network (blue) with a higher crosslinking density and higher elastic modulus, compared to the crosslinking between the same thiol and a dialkene linker (green). Images reproduced with from [101]. Copyright 2010 ACS.

Photo-initiated thiol-alkyne addition reactions were reported more than half a century ago but not used for materials development until recently, and received a lot less attention than other photocrosslinking reactions [84,100]. Compared to the 1:1 stoichiometry of the thiol-ene reaction, a thiol-yne addition utilizes each alkyne group to react with two thiols groups, where a vinylene sulfide intermediate is first formed and bears higher reactivity than the starting alkyne toward the addition of thiols (Figure 18). Theoretically, thiol-yne reactions result in higher crosslink density of polymer networks than thiol-ene hydrogels, and have been verified by their high conversion rates under ambient humidity and atmospheric oxygen conditions [101]. In early demonstrations of thiol-yne crosslinked hydrogels by Fairbanks et al. and Chan et al., uniform networks were produced from multi-armed macromonomers presenting thiol and alkyne groups [101,102]. The geometry of macromonomers exerted an effect on the reactivity of thiol and alkyne groups toward crosslinking, observed as the decreased network formation with increased branchness of macromonomers (multiplicity of arms) [102]. Unreacted thiol and alkyne groups on the hydrogel network can be used for incorporation of desired chemical functionalities either during crosslinking or post-gelation [103,104].

**Figure 19:**
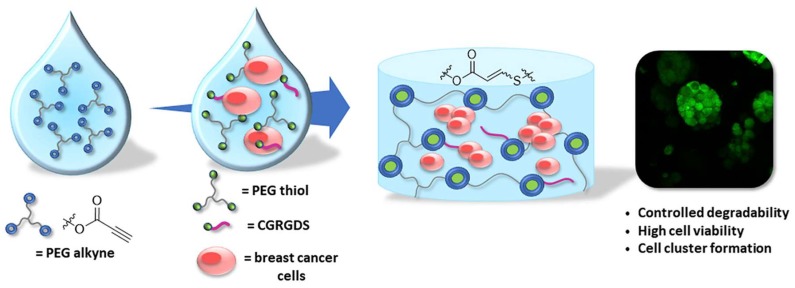
The non-radical nucleophilic addition reaction between multi-armed PEG-thiol and PEG-alkyne produced vinylene sulfide-linked hydrogel for encapsulation of MCF-7 breast cancer cells. An extracellular matrix mimicking peptide CGRGDS is incorporated to the gel through the same type of addition between the thiol group in the cysteine and PEG-alkyne during the network formation. The hydrogel is degradable through hydrolysis of the esters in PEG-alkyne to allow cell growth and formation of cell clusters. Images reproduced with permission from [107]. Copyright 2018 Elsevier.

As an alternative to the photo-initiated thiol-yne reaction, a non-radical thiol-yne addition was reported by Truong and Dove as a base-catalyzed nucleophilic addition reaction between thiols and electrophilic alkynes to produce vinylene sulfide [105]. This reaction has been shown to be efficient for crosslinking thiol- and alkyne-containing PEG macromonomers in PBS solutions at pH 7.4 [106], and applied to the formation of ECM-mimicking hydrogels as a model system for supporting cancer cell growth (Figure 19) [107].

A unique capacity of photo-initiated crosslinking methods (as well as degradation) mentioned above is to allow temporal and spatial control over the hydrogel structure and properties in order to generate complex bioactive scaffolds [108,109,110]. For instance, the thiol-ene photocrosslinking was used to fabricate ultrathin, micro-patterned hydrogel films on solid substrates and 3D-patterned peptide-presenting hydrogels with tunable crosslinking density and biochemical cues for cell encapsulation [92,111,112,113,114]. Again, as new methods for photo crosslinking advance toward the use of light in the visible and near IR range [78,115] and initiators with low toxicity [95,116,117], these hydrogels will find even wider applications in developing responsive biomaterials and creating complex 3D microenvironments for tissue engineering.

## 5. Summary and Perspectives

Covalently crosslinked hydrogels have structure stability and biomechanical properties that are tunable in a large range. Chemoselective crosslinking strategies are necessary for developing injectable, in situ-formed hydrogels in contact with the human body and encapsulated therapeutics. Although not completely bio-orthogonal, the thiol group is one of the most utilized functionalities for selective crosslinking of polymers, producing hydrogels through a diversity of chemical reactions, as discussed in this review. Kinetic control is necessary to limit the unwanted cross-reaction of polymers with a system of study and further improve the chemoselectivity and compatibility of the thiol-mediated hydrogel crosslinking in situ. It should be noted that, when thiol-containing polymers are present at high concentrations, disulfide formation among thiols is unavoidable and always results in additional crosslinking during and after the initial formation of hydrogel by a different reaction [35,118], which may make it difficult to precisely control the crosslinking degree of gel networks.

So far, studies of hydrogel material have been focused on bulk characteristics such as the rate of gelation, and the mechanical and/or biological properties, but the fundamental understanding of polymer crosslinking at the molecular level has not received adequate attention. For instance, during hydrogel network formation, the functionalities of the polymer backbone that are not directly involved in crosslinking reactions can gather and compose a local environment, influencing the continuation of crosslinking by H-bonding and acid–base catalysis (not only limited to thiol-based reactions). While the PEG backbone may be considered to be “inert” or “nondisturbing” to many crosslinking reactions, other types of polymers need to be examined depending on the specific crosslinking reaction used [119]. Investigation of the molecular interaction within the 3D network is challenging and requires the use of advanced instrumentation and computer-aided molecular simulation. The knowledge from future studies in this area will provide guidance for the design and selection of polymers and chemoselective strategies in applications of chemically crosslinked hydrogels in biomedical research and clinical practice. 

## Figures and Tables

**Table 1:** Thiol-mediated reactions for hydrogel crosslinking in situ.

Reaction	Advantages	Limitations
Disulfide Formation	Produces disulfide links that break down in a reductive environment Suitable for developing smart gels responsive to glutathione and other reducing agents	Slow gelation Relatively low selectivity When the thiol-disulfide exchange method is used, by-products are released.
Native Chemical Ligation	Catalyst-free Generates thiol-presenting networks for structure modifications	Low-molecular-weight thiol by-product released
Thiol-Epoxy Reaction	Catalyst-free No organic by-product released	Relatively slow gelation, high concentrations of reactants needed
Michael-Type Additions	Catalyst-free Rapid gelation and high crosslinking degree No by-product released	Possible cross-reactivity with amino groups Nonuniform crosslinking at the microscale Some crosslinks are unstable.
Photo-initiated Thiol-Ene and Thiol-Yne Additions	Fast gelation and tunable crosslinking density Allow spatial and temporal control of gel formation No by-product released	Require the use of photo-initiators that may be cytotoxic UV light can cause cellular damage.
